# Supplementary material for: The contribution of PARP1, PARP2 and poly(ADP-ribosyl)ation to base excision repair in the nucleosomal context
Source: Sci Rep. 2021 Mar 1;11:4849. doi: 10.1038/s41598-021-84351-1 (PMC7921663; doi:10.1038/s41598-021-84351-1)
Supplement: Supplementary file 1 — Supplementary Information. [file 41598_2021_84351_MOESM1_ESM.docx]

**SUPPLEMENTARY INFORMATION**

**The contribution of PARP1, PARP2 and poly(ADP-ribosyl)ation to base excision repair in the nucleosomal context**

Kutuzov M.M. ^1†^, Belousova E.A. ^1†^, Kurgina T.A. ^1,2^, Ukraintsev A.A. ^1,2^, Vasil’eva I.A. ^1^, Khodyreva S.N. ^1^, Lavrik O.I. ^1,2^*

^1^Institute of Chemical Biology and Fundamental Medicine, SB RAS, Novosibirsk, Russia

^2^Novosibirsk State University, Novosibirsk, Russia

^†^These authors contributed equally to this work

*To whom correspondence should be addressed. lavrik@niboch.nsc.ru


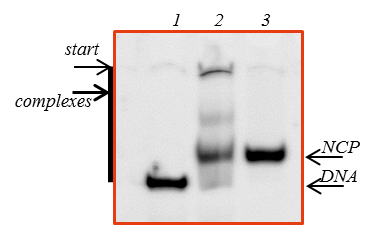


**Figure S1.** Electrophoretic mobility of 5′-FAM–labelled products after NCP reconstruction from model FAM-DNA and histone octamers in a 4% polyacrylamide gel under non-denaturing conditions. Lane 1: free DNA; lane 2: a sample obtained by mixing of DNA and the histone octamer at a 1:1 ratio in low-salt reaction buffer; lane 3: the NCP assembled by gradient dialysis at the 1:1 ratio of DNA to the histone octamer. NCP: nucleosome core particle, DNA: naked DNA, complexes: complexes of DNA with histone octamers.

**
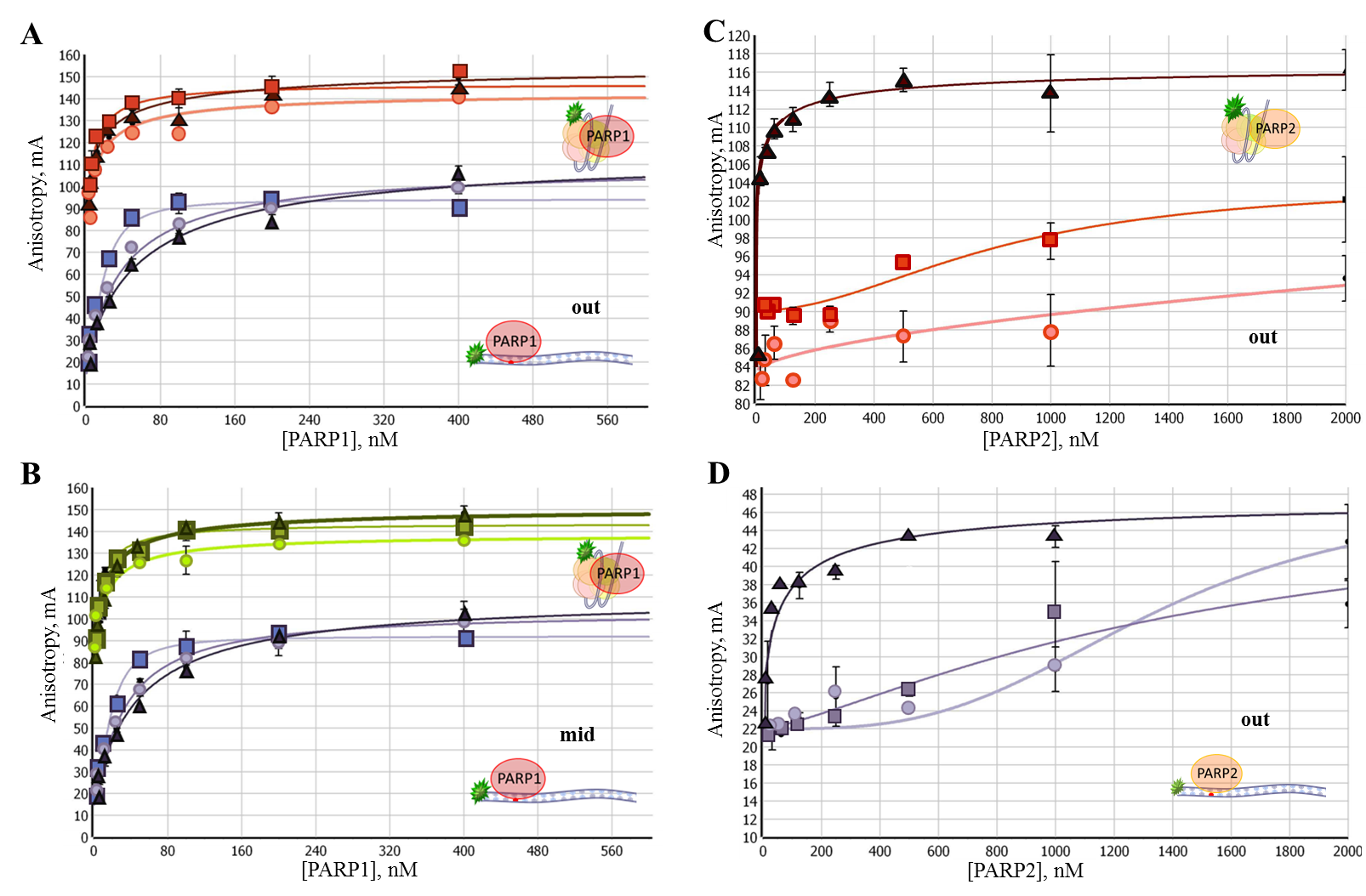
**

**
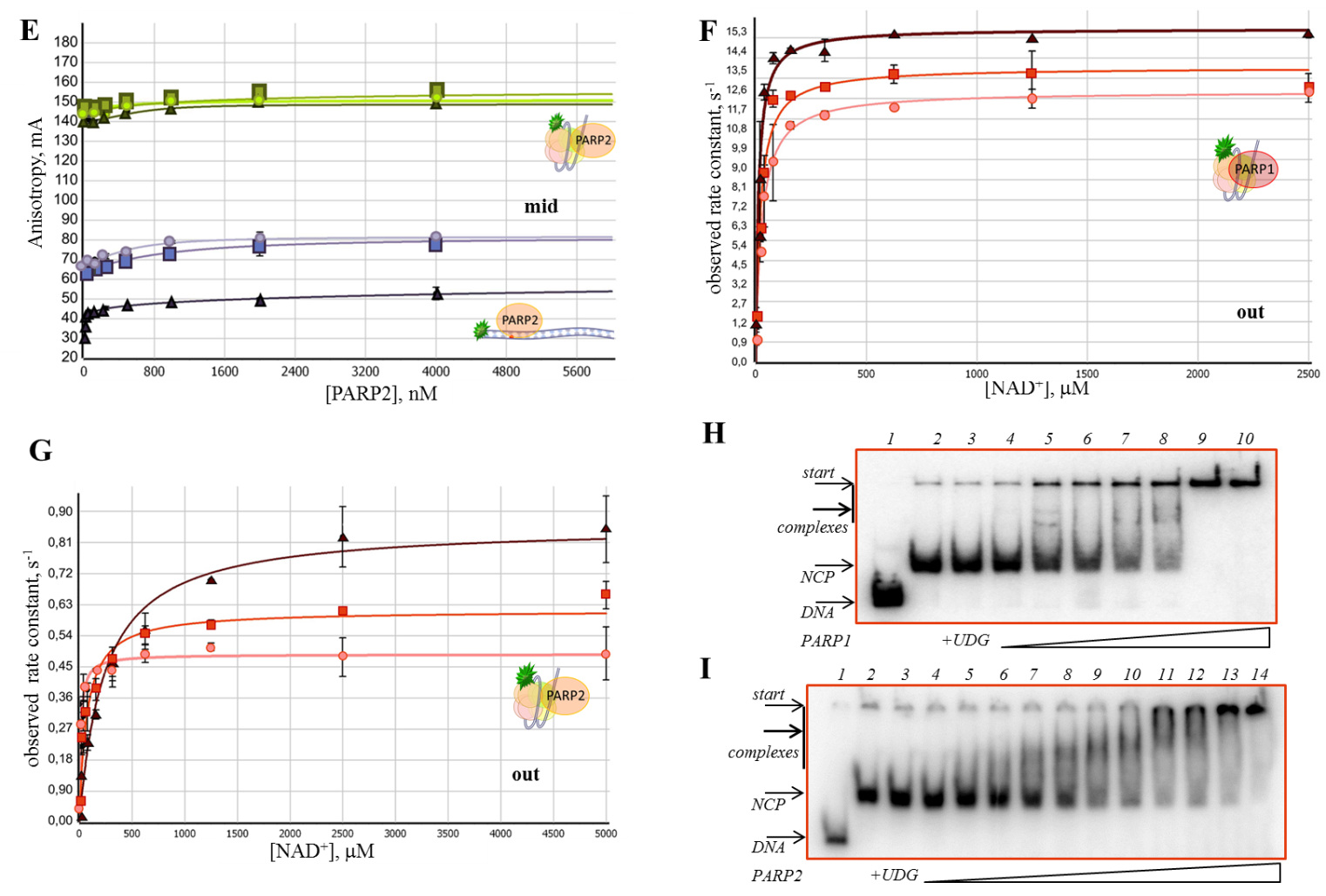
**

**Figure S2.** The affinity of PARP1 (A and B) and PARP2 (C, D and E) for native (circles), AP- (squares) or gap-NCP (triangles) or DNA as measured by fluorescence anisotropy using 100 nM 5′-FAM–labelled NCP with an outward-oriented lesion (out-NCP; panels A, C and D) and the NCP with middle-oriented damage (mid-NCP; panels B and E). (F and G) Observed rate constant changes for protein–nucleic acid complexes of out-NCP with PARP1 (F) or PARP2 (G) under PARylation conditions. The reaction mixtures contained 30 nM 5′-FAM–labelled out-NCP, 100 nM PARP1 or 1000 nM PARP2 at various concentrations of NAD^+^. In all the graphs, the experimental data on NCP substrates correspond to the red or yellowish-green curves; the DNA substrates are characterised by black, reddish-violet or violet curves. The data are presented as an average of at least three independent experiments. (H) EMSA analysis of the protein–nucleic acid complexes of PARP1 with 5′[^32^P]labelled AP-NCP. Lane 1: 147 nt DNA, lane 2: native NCP, lanes 3: AP-NCP, lanes 4–10: complexes of PARP1 with AP-NCP. (I) EMSA analysis of the protein–nucleic acid complexes of PARP2 with 5′[^32^P]labelled AP-NCP. Lane 1: 147 nt DNA, lane 2: native NCP, lane 3: AP-NCP, lanes 4–14: complexes of PARP2 with AP-NCP.

|  |
| --- |
| **Figure S3**. The overall level of PAR synthesized by PARP1 or PARP2 in the presence of PARG. The efficacy of PAR synthesis was evaluated as the amount of a poly(ADP-ribose) synthesised by PARP1 or PARP2 using [^32^P]labelled NAD^+^ as a precursor. The reaction (in 90 μl) was initiated by the addition of (i) 50 nM PARP1 (blue curve); (ii) 50 nM PARP1 and 50 nM PARG (dashed blue curve); (iii) 100 nM PARP2 (green curve); (iv) 100 nM PARP2 and 50 nM PARG (dashed green curve) to a solution of 50 nM 5′-FAM–labelled NCP in reaction buffer with 5 mM MgCl_2_ and 1,000 μM NAD^+^ containing 1 μM [^32^P]labelled NAD^+^. The reaction was carried out at 37°C for 0, 0.5, 1, 2, 3, 5, 10 and 25 min and stopped by placing the aliquots of the reaction mixture on paper filters (Whatman-1) soaked with a 5% solution of trichloroacetic acid. The filters were washed four times with 5% trichloroacetic acid, then with 90% ethanol, and air-dried. The filters were subjected to autoradiography for quantitation on the Typhoon imaging system (GE Healthcare Life Sciences), and the quantity of the radiolabel incorporated into the acid-insoluble fraction was analysed using the Quantity One software (Bio-Rad). The quantitative data were analysed in Microsoft Excel 2010 and are presented in histograms as the mean ± SD. |

|  |
| --- |
|  |
| **Figure S4.** The overall level of degradation of PAR synthesized by PARP1 or PARP2. The efficacy of PAR hydrolysis was evaluated as the amount of poly(ADP-ribose) initially synthesised by PARP1 or PARP2 using [^32^P]labelled NAD^+^ as a precursor. The 90 μl reaction was initiated by the addition of (i) 50 nM PARP1 (blue curves); (ii) 100 nM PARP2 (green curves) to a solution of 50 nM 5′‑FAM–labelled NCP in reaction buffer with 5 mM MgCl_2_ and 1,000 μM NAD^+^ containing 1 μM [^32^P]labelled NAD^+^. The reaction was carried out at 37°C for 15 min, and then the reaction mixtures were placed on ice and divided into two equal portions, one of which was supplemented with 15 mM EDTA. After that, 50 nM PARG was added to both portions with subsequent incubation at 37°C for 0, 0.5, 1, 2, 3, 5, 10 or 25 min, and the reaction was stopped by placing the aliquots of the reaction mixture on paper filters (Whatman-1) soaked with a 5% solution of trichloroacetic acid. The filters were washed four times with 5% trichloroacetic acid, then with 90% ethanol, and air-dried. The filters were subjected to autoradiography for quantitation on the Typhoon imaging system (GE Healthcare Life Sciences), and the quantity of the radiolabel incorporated into the acid-insoluble fraction was analysed using Quantity One software (Bio-Rad). The quantitative data were analysed in Microsoft Excel 2010 and are presented in histograms as the mean ± SD. |

|  |
| --- |
| **Figures S5**. The kinetics of AP site cleavage in AP-NCP by APE1 in the presence of PARP1 and PARG. APE1 efficacy was evaluated as the amount of an AP site cleavage product relative to all forms of DNA in a lane, expressed as a percentage. The 70 μl reaction was initiated by the addition of 0.03 μM APE1 (blue curve) to a solution of 0.1 μM 5'-FAM–labelled AP-substrate in reaction buffer with 5 mM MgCl_2_. The reaction was carried out at 37°C for 0, 1, 3, 5, 10 or 15 min and stopped by the addition of 20 mM methoxyamine with 15 mM EDTA and incubated for 30 min on ice. The reaction mixtures were supplemented with loading buffer consisting of 7 M urea and 50 mM EDTA. For further experiments, according to the same experimental scheme, 50 nM PARG (dashed blue curve); 100 nM PARP1 (red curve); 100 nM PARP1 and 50 nM PARG (dashed red curve); 100 nM PARP1 and 1000 µM NAD^+^ (green curve); or 100 nM PARP1, 1000 µM NAD^+^ and 50 nM PARG (dashed green curve) were added. All reaction products were separated by 10% polyacrylamide gel electrophoresis and analysed using Quantity One software (Bio-Rad). The quantitative data were analysed in Microsoft Excel 2010 and are presented in histograms as the mean ± SD. All presented data were obtained using out-NCP. |

|  |
| --- |
| **Figure S6.** The kinetics of AP site cleavage by APE1 in AP-NCP in the presence of PARP2 and PARG. APE1 efficacy was evaluated as the amount of an AP site cleavage product relative to all forms of DNA in a lane, expressed as a percentage. The 70 μl reaction was initiated by the addition of 0.03 μM APE1 (blue curve) to a solution of 0.1 μM 5'-FAM–labelled AP-NCP in reaction buffer with 5 mM MgCl_2_. The reaction was carried out at 37°C for 0, 1, 3, 5, 10 or 15 min and stopped by the addition of 20 mM methoxyamine with 15 mM EDTA and incubated for 30 min on ice. The reaction mixtures were supplemented with loading buffer consisting of 7 M urea and 50 mM EDTA. For further experiments, in accordance with the same experimental scheme, 50 nM PARG (dashed blue curve); 500 nM PARP2 (red curve); 500 nM PARP2 and 50 nM PARG (dashed red curve); 500 nM PARP2 and 1000 µM NAD^+^ (green curve); or 500 nM PARP2, 1000 µM NAD^+^ and 50 nM PARG (dashed green curve) were added. All reaction products were separated by 10% polyacrylamide gel electrophoresis and analysed using Quantity One software (Bio-Rad). The quantitative data were analysed in Microsoft Excel 2010 and are presented in histograms as the mean ± SD. All presented data were obtained with out-NCP. |


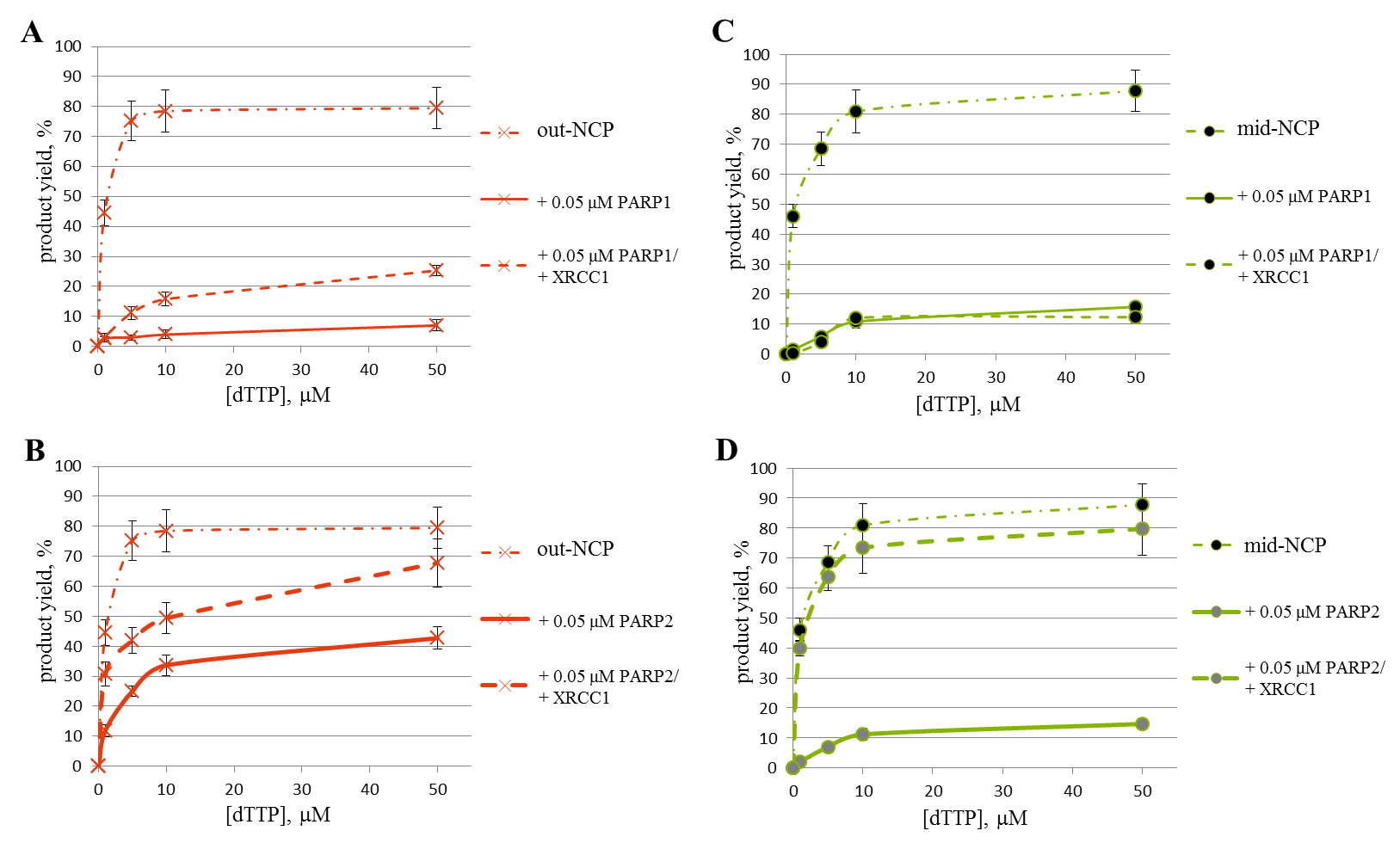


**Figure S7.** The activity of Polβ towards substrates gap-NCP by itself (dot-and-dash red or yellowish-green curves) and in the presence of PARP1 or PARP2 and XRCC1. The kinetic curves of dTMP incorporation were obtained using outward-oriented (A, B) or midward-oriented (C, D) gap-NCP in the presence of PARP1 (solid red or yellowish-green curves), PARP1 and XRCC1 (dashed red or yellowish-green curves), PARP2 (solid red or yellowish-green curves) or PARP2 and XRCC1 (dashed red or yellowish-green curves). For details see the ‘Materials and Methods’ section.

|  |
| --- |
|  |
| **Figure S8**. The influence of PARG on the yield of the reaction products of DNA synthesis catalysed by Polβ using gap-NCP. The efficacy of the DNA synthesis was evaluated as the amount of the dTMP incorporated product relative to the initial gap-containing NCP form, expressed as a percentage. The reaction (in 10 μl) was initiated by the addition of 2.5 nM Polβ (columns "b" and "b+nad") to a solution of 0.1 μM 5′-FAM–labelled gap-NCP and 10 µM dTTP in reaction buffer with 5 mM MgCl_2_. The reaction was carried out at 37°C for 3 min and stopped by the addition of loading buffer consisting of 7 M urea and 50 mM EDTA. For further experiments, according to the same experimental scheme, 50 nM PARG (+pg), 50 nM PARP1 (+p1), and/or 1, 10, 100 or 1000 µM NAD^+^ (nad1, nad2, nad3 or nad4, respectively) were added. The last four bars were calculated from the following experiments. The 10 μl reaction was initiated by the addition of 2.5 nM Polβ and 50 nM PARP1 or 50 nM PARP1 with 1, 10, 100 or 1000 µM NAD^+^ to a solution of 0.1 μM 5′-FAM–labelled gap-NCP and 10 µM dTTP in reaction buffer with 5 mM MgCl_2_ and incubated at 37°C for 3 min. After that, the reactions were supplemented with 50 nM PARG and incubated for additional 3 min at 37°C. All the same experiments were conducted in the presence of 4 nM XRCC1 (striped bars). All reaction products were separated by 10% polyacrylamide gel electrophoresis and analysed using Quantity One software (Bio-Rad). The quantitative data were analysed in Microsoft Excel 2010 and are presented in histograms as the mean ± SD. All presented data were obtained with out-NCP. |

|  |
| --- |
|  |
| **Figure S9.** The influence of PARG on the yield of the reaction products of DNA synthesis catalysed by Polβ using gap-NCP. The efficacy of the DNA synthesis was evaluated as the amount of the dTMP incorporation product relative to the initial gap-containing NCP form, expressed as a percentage. The reaction (in 10 μl) was initiated by the addition of 2.5 nM Polβ (columns "b" and "b+nad") to a solution of 0.1 μM 5′-FAM–labelled gap-NCP and 10 µM dTTP in reaction buffer with 5 mM MgCl_2_. The reaction was carried out at 37°C for 3 min and stopped by the addition of loading buffer consisting of 7 M urea and 50 mM EDTA. For further experiments, in accordance with the same experimental scheme, 50 nM PARG (+pg), 50 nM PARP2 (+p1), and/or 1, 10, 100 or 1000 µM NAD^+^ (nad1, nad2, nad3 or nad4, respectively) were added. The last four bars were calculated from the following experiments. The 10 μl reaction was initiated by the addition of 2.5 nM Polβ and 50 nM PARP2 or 50 nM PARP2 with 1, 10, 100 or 1000 µM NAD^+^ to a solution of 0.1 μM 5′-FAM–labelled gap-NCP and 10 µM dTTP in reaction buffer with 5 mM MgCl_2_ and incubated at 37°C for 3 min. After that, the reactions were supplemented with 50 nM PARG and incubated for additional 3 min at 37°C. All the same experiments were conducted in the presence of 4 nM XRCC1 (striped bars). All reaction products were separated by 10% polyacrylamide gel electrophoresis and analysed using Quantity One software (Bio-Rad). The quantitative data were analysed in Microsoft Excel 2010 and are presented in histograms as the mean ± SD. All presented data were obtained using out-NCP. |


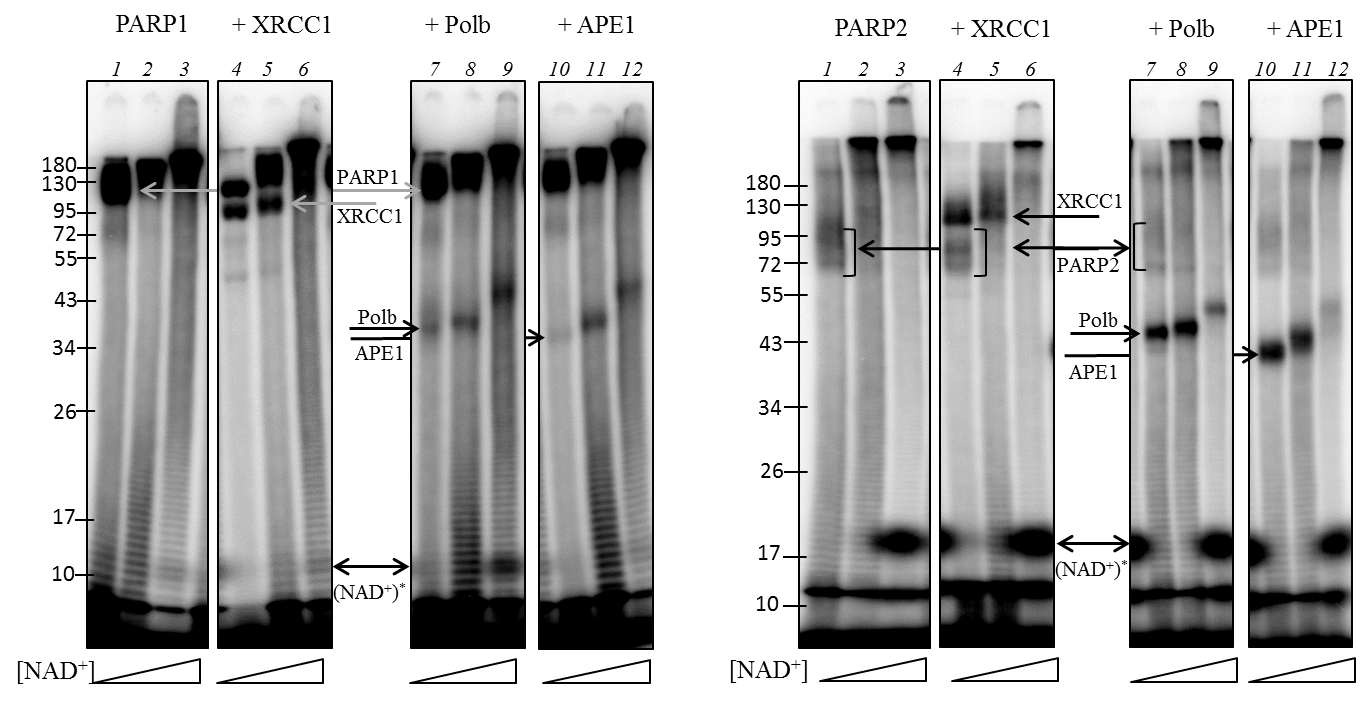


**Figure S10.** PARylation of XRCC1, Polβ and APE1 by PARP1 (left panel) and by PARP2 (right panel) using [^32^P]labelled NAD^+^. Lanes 1–3: autoPARylation of PARP, lanes 4–6: PARylation of XRCC1, lanes 7–9: PARylation of Polβ, and lanes 10–12: PARylation of APE1. The molecular masses in kilodaltons are indicated on the left. The reactions were started by the addition of 1, 10 or 100 μM NAD^+^ with an isotopic dilution of 1:0, 1:9 or 1:99, respectively, towards the solution of 0.1 μM 34 nt 5′-phosphorylated one-window gapped DNA duplex in complex with 0.5 μM PARP1 or PARP2 and 1 μM XRCC1, Polβ or APE1 in reaction buffer with 2 mM MgCl_2_. The reaction was allowed to proceed for 15 min at 37°C and was stopped by the addition of Laemmli buffer. The products were separated by 12% polyacrylamide gel electrophoresis, dried and subjected to autoradiography using the Typhoon imaging system (GE Healthcare Life Sciences) and analysed in the Quantity One software (Bio-Rad).


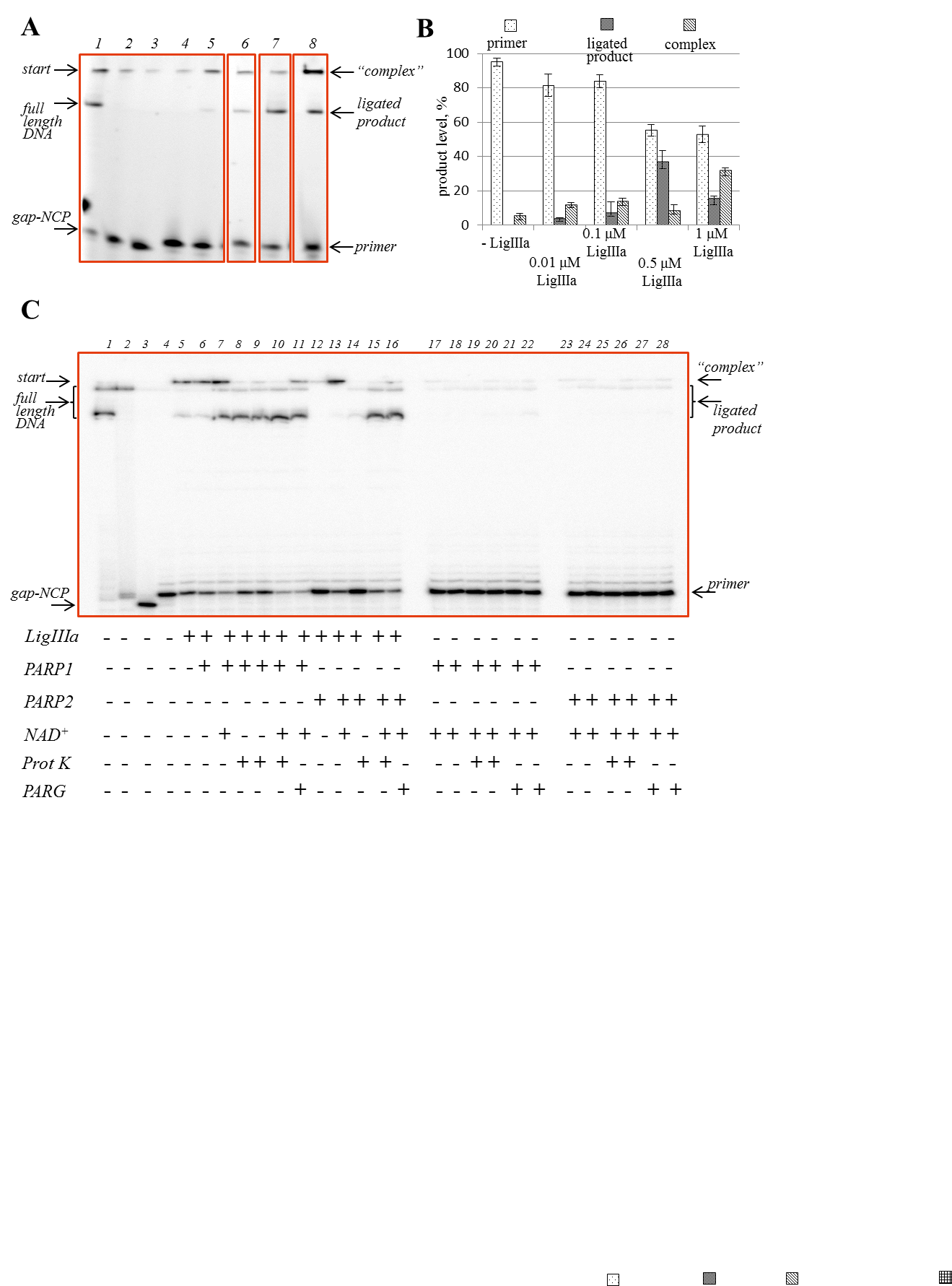


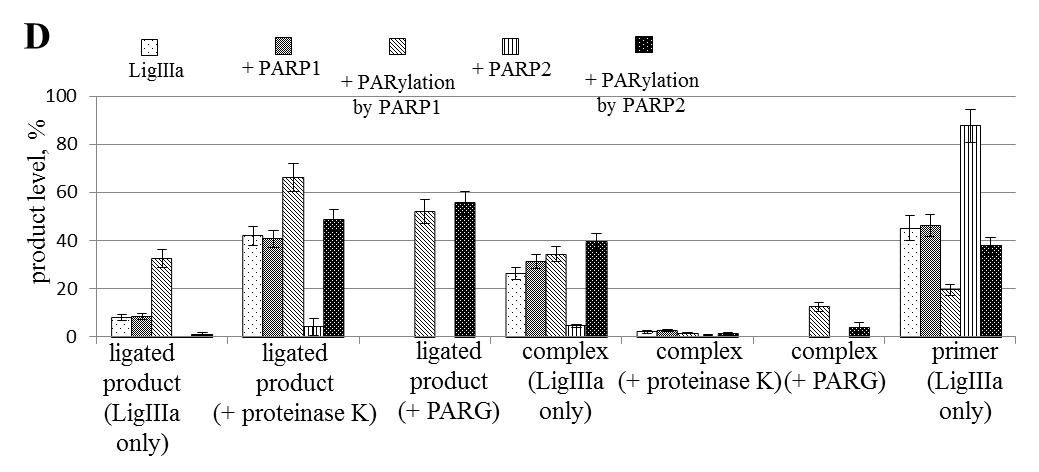


**Figure S11.** The dependence of the LigIIIα activity on substrate ‘nicked out-NCP’. Product separation (A) and quantitative analysis (B) of the sealing reaction at different LigIIIα concentrations. The reaction procedures are described in the ‘Materials and Methods’ section. (A) Lane 1: native out-NCP; lane 2: out-NCP incubated with UDG; lane 3: out-NCP incubated with UDG and APE; lane 4: out-NCP incubated with UDG, APE, Polβ and dTTP resulting in nicked out-NCP; and lanes 5–8: nick sealing in the presence of 0.01, 0.1, 0.5 or 1 μM of LigIIIα. (B) The bars correspond to the level of the initial substrate (dotted), ligated products (hatched) and undenatured complexes (striped) in lines 4–8, respectively.

The product separation (C) and quantitative analysis (D) of the sealing of nicked out-NCP by LigIIIα with PARP1 or PARP2 and PARylation in the presence of XRCC1. The reaction procedures are described in the ‘Materials and Methods’ section. (C) Lane 1: native out-NCP; lane 2: out-NCP incubated with UDG; lane 3: out-NCP incubated with UDG and APE; lane 4: out-NCP incubated with UDG, APE, Polβ and dTTP resulting in nicked out-NCP; lanes 5–16: nick sealing in the presence of 0.5 μM LigIIIα (lanes 5 and 8) and 0.1 μM PARP1 (lanes 6 and 9) or PARP2 (lanes 12 and 14) without or with 100 μM NAD^+^ (lanes 7, 10, 11 and 13, 15, 16, respectively). Lanes 8–10, 14–15, 19–20 and 26–26 correspond to lanes 5–7, 12–13, 17–18 and 23–24 with additional treatment (proteinase K). Lanes 11, 16, 21–22 and 27–28 correspond to lanes 7, 13, 17–18 and 23–24 with additional treatment (PARG). Lanes 17–22: reaction mixtures containing nicked NCP with 0.1 μM PARP1 and 1 or 100 μM NAD^+^ in pairs. Lanes 23–28: reaction mixtures containing nicked NCP with 0.1 μM PARP2 and 1 or 100 μM NAD^+^ in pairs. (D) The bars correspond to the level of the indicated product obtained in the reactions with LigIIIα (dotted) only or in the presence of PARP1 (hatched) or PARP2 (vertically stripped) or under PARylation by PARP1 (striped) or by PARP2 (black with white dots). All the data are presented as an average of at least three independent experiments.

Table S1. *The affinity of PARP1 and PARP2 for native, AP-NCP or gap-NCP according to the EMSA*

| *K*_d_, nM, for substrates with  an outward-oriented lesion | | | |
| --- | --- | --- | --- |
|  | NCP | | |
|  | native | AP site | gap |
| PARP1 | 65±3 | 61±5 | 38±4 |
| PARP2 | 192±10 | 130±4 | 57±8 |

**The original images of full-length gels to all figures**


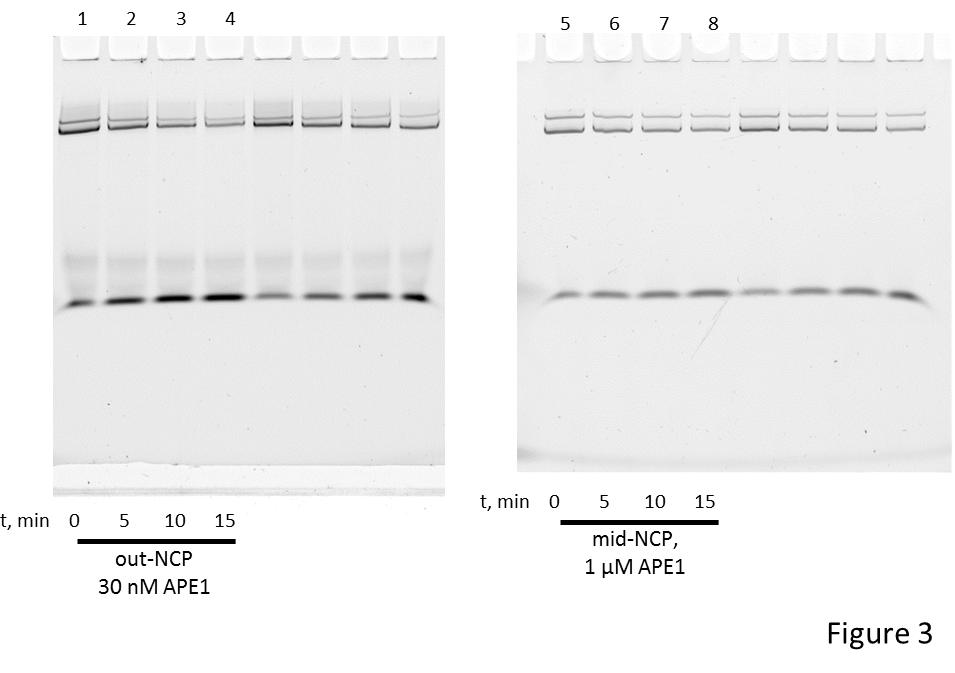


**Figure 3.** The kinetic assay of APE1 activity towards an outward- or midward-oriented AP site in the NCP context in the presence of PARP1 or PARP2. (f) Separation of the products (on a 10% denaturing polyacrylamide gel) of the reaction of 0.1 μM 5′-FAM–labelled AP-NCP with APE1 under the indicated experimental conditions. In all cases, the APE concentrations of 0.03 and 1 μM were chosen for the reaction conditions involving substrates out-NCP and mid-NCP, respectively.


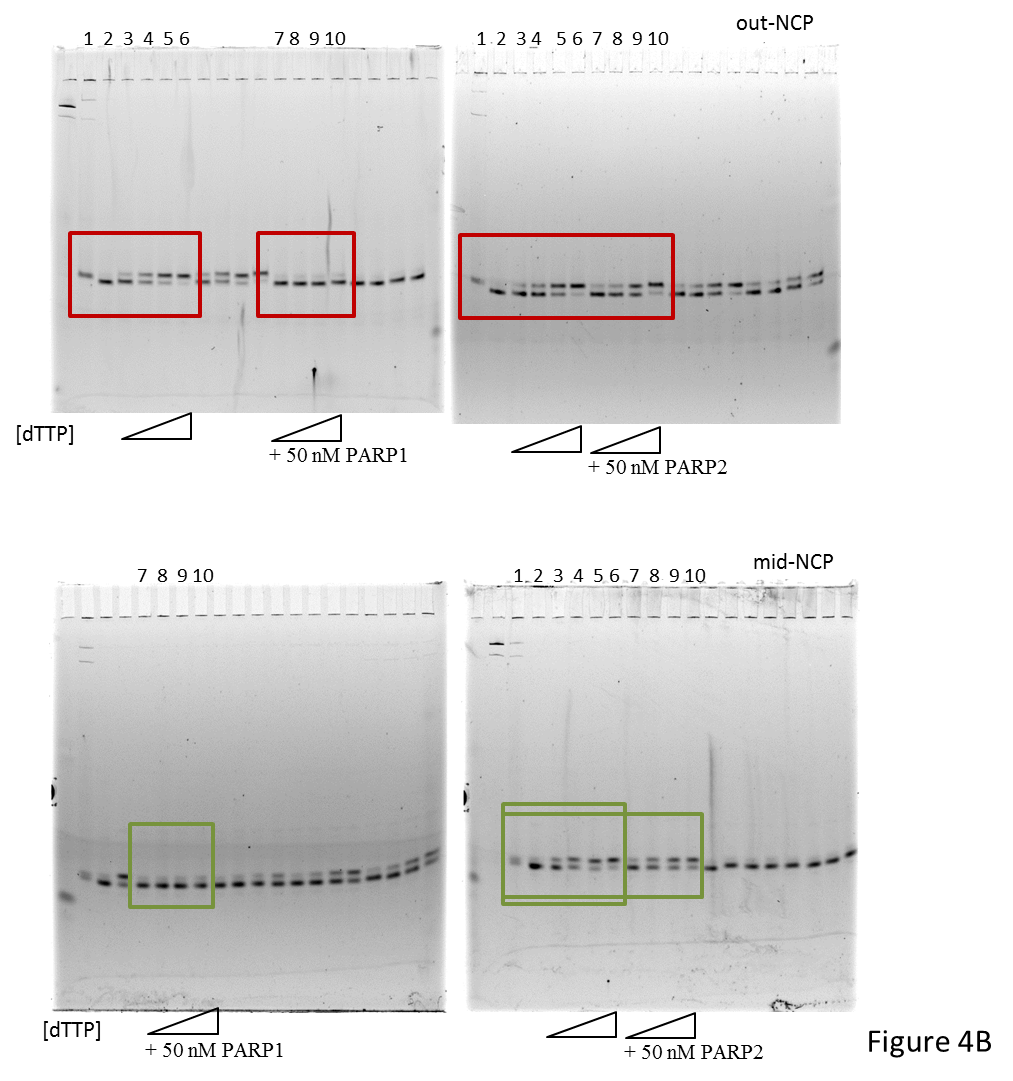


**Figure 4.** The activity of Polβ towards gap-NCP substrates by itself and during PARylation catalysed by PARP1 or PARP2. (b) The reaction products of dTMP incorporation by Polβ (lanes 3–6) in the presence of PARP1 (left part) or PARP2 (right part, lanes 7–10) when outward- (upper panel) or midward-oriented (lower panel) 5′-FAM–labelled gap-NCP was employed. Lane 1: substrate AP-NCP, lane 2: substrate gap-NCP. In all cases, Polβ concentrations of 2.5 and 50 nM were chosen as the reaction conditions for substrates out-NCP and mid-NCP, respectively. The reaction products were separated on a 15% denaturing polyacrylamide gel.


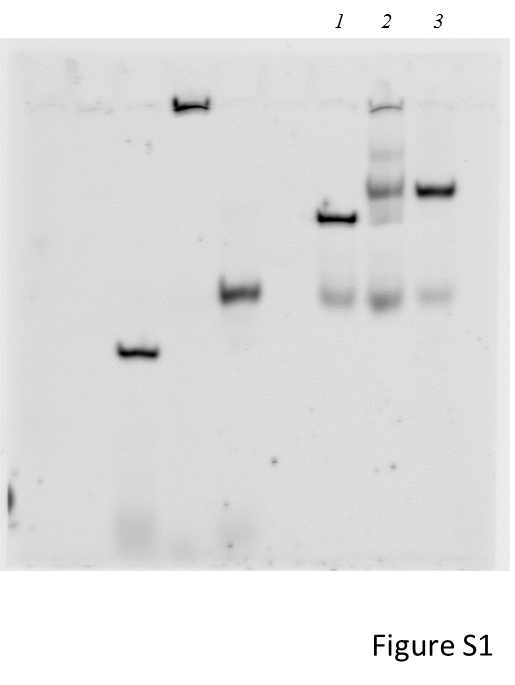


**Figure S1.** Electrophoretic mobility of 5′-FAM–labelled products after NCP reconstruction from model FAM-DNA and histone octamers in a 4% polyacrylamide gel under non-denaturing conditions. Lane 1: free DNA; lane 2: a sample obtained by mixing of DNA and the histone octamer at a 1:1 ratio in low-salt reaction buffer; lane 3: the NCP assembled by gradient dialysis at the 1:1 ratio of DNA to the histone octamer. NCP: nucleosome core particle, DNA: naked DNA, complexes: complexes of DNA with histone octamers.


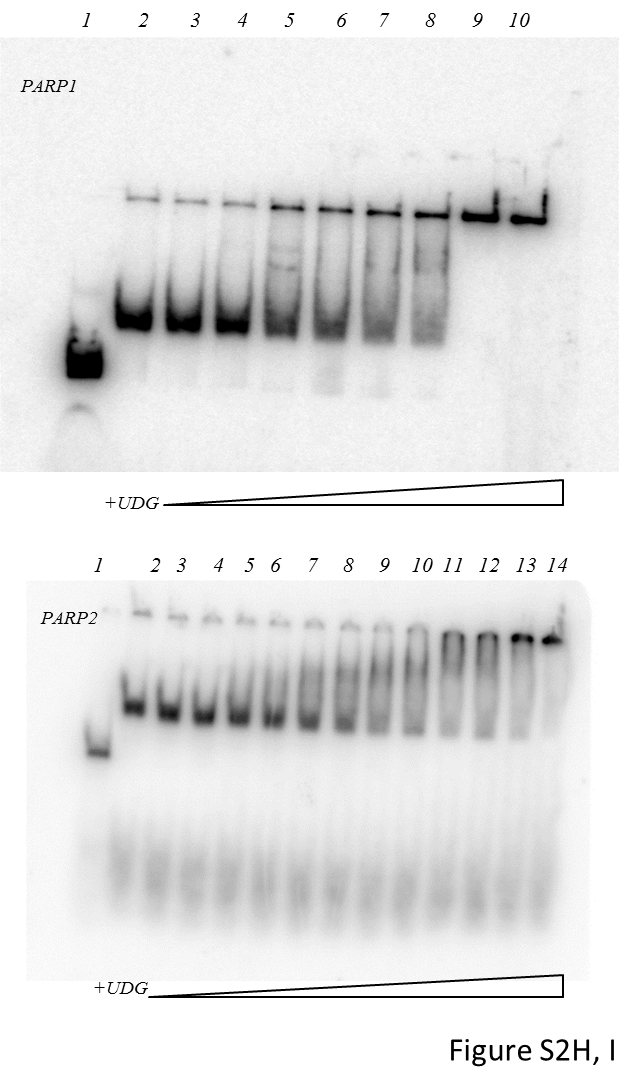


**Figure S2.** The affinity of PARP1 and PARP2 for AP-NCP.(H) EMSA analysis of the protein–nucleic acid complexes of PARP1 with 5′[^32^P]labelled AP-NCP. Lane 1: 147 nt DNA, lane 2: native NCP, lanes 3: AP-NCP, lanes 4–10: complexes of PARP1 with AP-NCP. (I) EMSA analysis of the protein–nucleic acid complexes of PARP2 with 5′[^32^P]labelled AP-NCP. Lane 1: 147 nt DNA, lane 2: native NCP, lane 3: AP-NCP, lanes 4–14: complexes of PARP2 with AP-NCP.


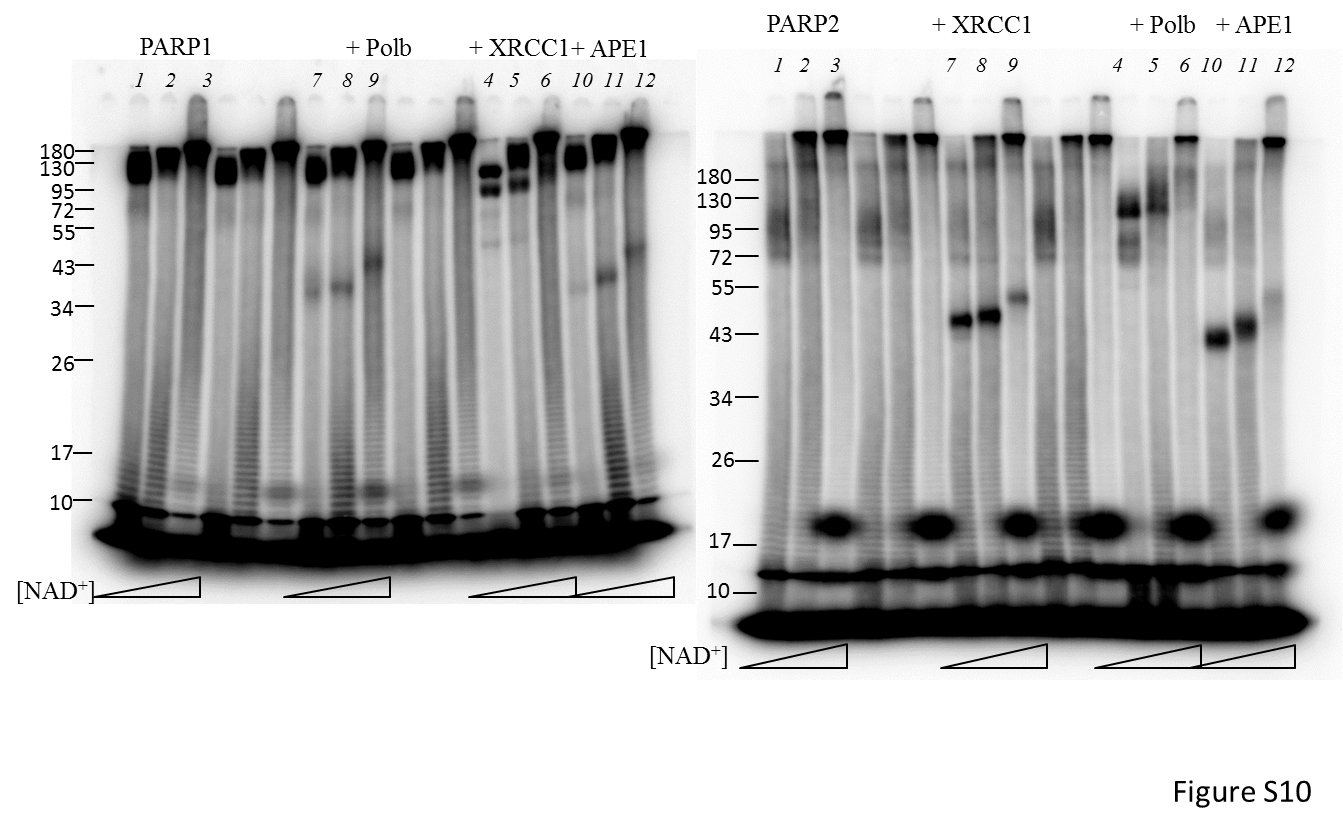


**Figure S10.** PARylation of XRCC1, Polβ and APE1 by PARP1 (left panel) and by PARP2 (right panel) using [^32^P]labelled NAD^+^. Lanes 1–3: autoPARylation of PARP, lanes 4–6: PARylation of XRCC1, lanes 7–9: PARylation of Polβ, and lanes 10–12: PARylation of APE1. The molecular masses in kilodaltons are indicated on the left. The reactions were started by the addition of 1, 10 or 100 μM NAD^+^ with an isotopic dilution of 1:0, 1:9 or 1:99, respectively, towards the solution of 0.1 μM 34 nt 5′-phosphorylated one-window gapped DNA duplex in complex with 0.5 μM PARP1 or PARP2 and 1 μM XRCC1, Polβ or APE1 in reaction buffer with 2 mM MgCl_2_. The reaction was allowed to proceed for 15 min at 37°C and was stopped by the addition of Laemmli buffer. The products were separated by 12% polyacrylamide gel electrophoresis, dried and subjected to autoradiography using the Typhoon imaging system (GE Healthcare Life Sciences) and analysed in the Quantity One software (Bio-Rad).


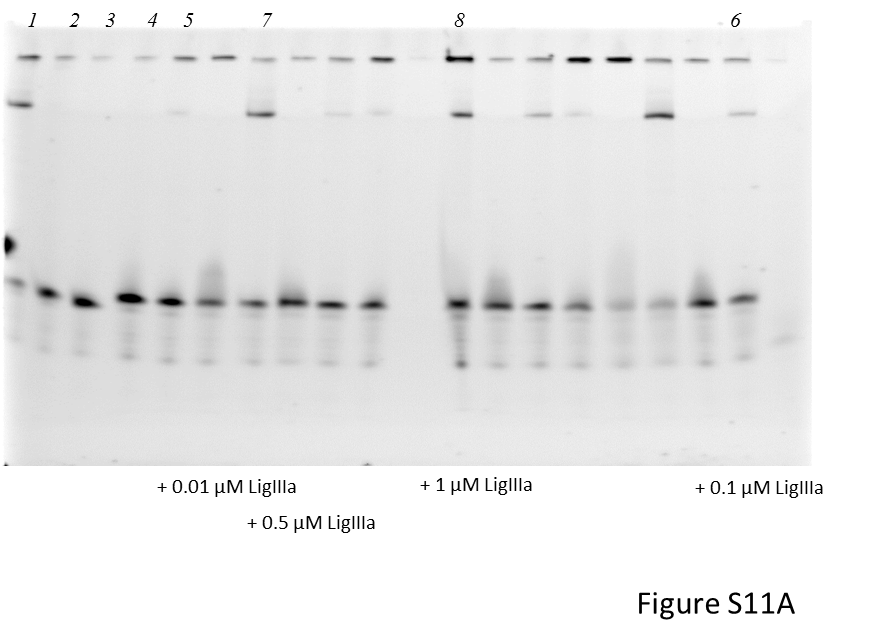


**Figure S11.** The dependence of the LigIIIα activity on substrate ‘nicked out-NCP’. Product separation (A) of the sealing reaction at different LigIIIα concentrations. The reaction procedures are described in the ‘Materials and Methods’ section. (A) Lane 1: native out-NCP; lane 2: out-NCP incubated with UDG; lane 3: out-NCP incubated with UDG and APE; lane 4: out-NCP incubated with UDG, APE, Polβ and dTTP resulting in nicked out-NCP; and lanes 5–8: nick sealing in the presence of 0.01, 0.1, 0.5 or 1 μM of LigIIIα.


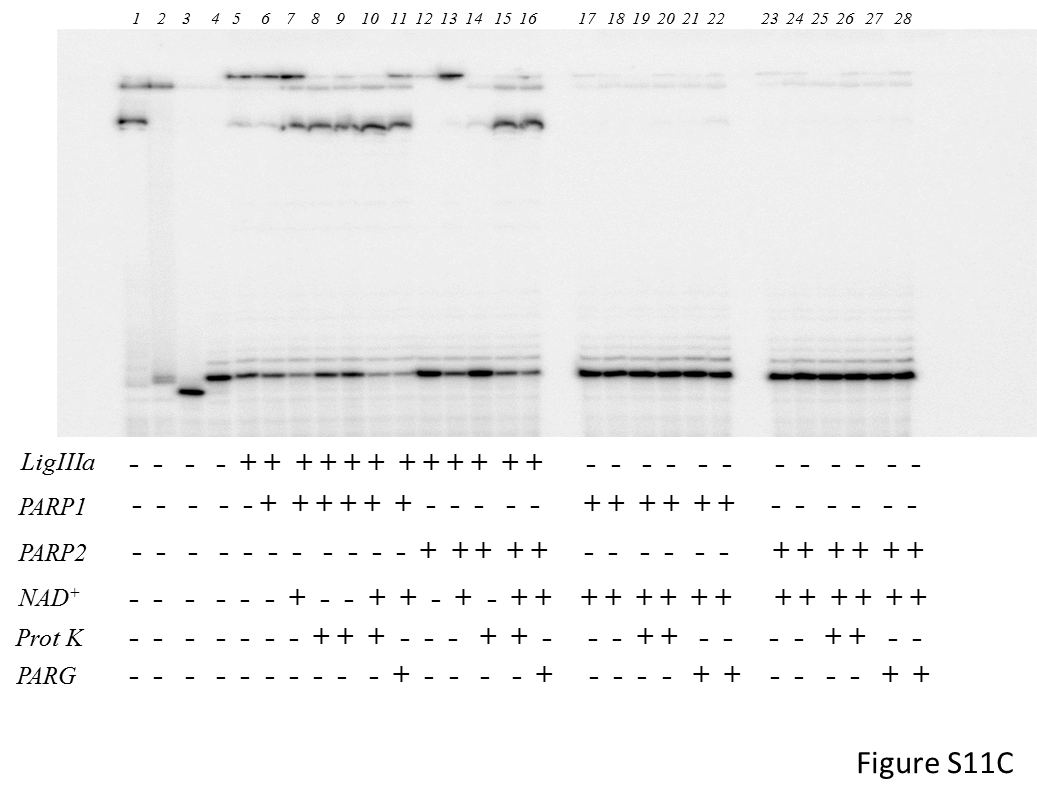


**Figure S11.** The dependence of the LigIIIα activity on substrate ‘nicked out-NCP’.

The product separation (C) of the sealing of nicked out-NCP by LigIIIα with PARP1 or PARP2 and PARylation in the presence of XRCC1. The reaction procedures are described in the ‘Materials and Methods’ section. (C) Lane 1: native out-NCP; lane 2: out-NCP incubated with UDG; lane 3: out-NCP incubated with UDG and APE; lane 4: out-NCP incubated with UDG, APE, Polβ and dTTP resulting in nicked out-NCP; lanes 5–16: nick sealing in the presence of 0.5 μM LigIIIα (lanes 5 and 8) and 0.1 μM PARP1 (lanes 6 and 9) or PARP2 (lanes 12 and 14) without or with 100 μM NAD^+^ (lanes 7, 10, 11 and 13, 15, 16, respectively). Lanes 8–10, 14–15, 19–20 and 26–26 correspond to lanes 5–7, 12–13, 17–18 and 23–24 with additional treatment (proteinase K). Lanes 11, 16, 21–22 and 27–28 correspond to lanes 7, 13, 17–18 and 23–24 with additional treatment (PARG). Lanes 17–22: reaction mixtures containing nicked NCP with 0.1 μM PARP1 and 1 or 100 μM NAD^+^ in pairs. Lanes 23–28: reaction mixtures containing nicked NCP with 0.1 μM PARP2 and 1 or 100 μM NAD^+^ in pairs.
